# A new allele conferring resistance to *Lysinibacillus sphaericus* is detected in low frequency in *Culex quinquefasciatus* field populations

**DOI:** 10.1186/s13071-016-1347-2

**Published:** 2016-02-04

**Authors:** Heverly Suzany Gouveia Menezes, Karlos Diogo de Melo Chalegre, Tatiany Patrícia Romão, Cláudia Maria Fontes Oliveira, Osvaldo Pompílio de-Melo-Neto, Maria Helena Neves Lobo Silva-Filha

**Affiliations:** Department of Entomology, Centro de Pesquisas Aggeu Magalhães-FIOCRUZ, Recife, PE 50740-465 Brazil; Department of Microbiology, Centro de Pesquisas Aggeu Magalhães-FIOCRUZ, Recife, PE 50740-465 Brazil

**Keywords:** Resistance, *cqm1* alleles, Binary toxin, PCR-screening

## Abstract

**Background:**

The Cqm1 α-glucosidase of *Culex quinquefasciatus* larvae acts as the midgut receptor for the binary toxin of the biolarvicide *Lysinibacillus sphaericus*. Mutations within the *cqm1* gene can code for aberrant polypeptides that can no longer be properly expressed or bind to the toxin, leading to insect resistance. The *cqm1*_*REC*_ and *cqm1*_*REC-2*_ alleles were identified in a laboratory selected colony and both displayed mutations that lead to equivalent phenotypes of refractoriness to *L. sphaericus. cqm1*_*REC*_ was first identified as the major resistance allele in this colony but it was subsequently replaced by *cqm1*_*REC-2*_, suggesting the better adaptive features of the second allele. The major aim of this study was to evaluate the occurrence of *cqm1*_*REC-2*_ and track its origin in field populations where *cqm1*_*REC*_ was previously identified.

**Methods:**

The screening of the *cqm1*_*REC-2*_ allele was based on more than 2000 *C. quinquefasciatus* larvae from five localities in the city of Recife, Brazil and used a multiplex PCR assay that is also able to identify *cqm1*_*REC*_. Full-length sequencing of the *cqm1*_*REC-2*_ and selected *cqm1* samples was performed to identify further polymorphisms between these alleles.

**Results:**

The *cqm1*_*REC-2*_ allele was found in field samples, specifically in two heterozygous individuals from a single locality with an overall frequency and distribution much lower than that observed for *cqm1*_*REC*_. The full-length sequences from these two *cqm1*_*REC-2*_ copies were almost identical to the *cqm1*_*REC-2*_ derived from the resistant colony but displayed more than 30 SNPs when compared with *cqm1* and *cqm1*_*REC*_. The *cqm1*_*REC*_ and *cqm1*_*REC-2*_ resistant alleles were found to be associated with two distinct sets of wild-type *cqm1* variants found in field populations.

**C**onclusions**:**

The *cqm1*_*REC-2*_ allele occurs in populations in Recife and was probably already present in the samples used to establish the laboratory resistant colony. The data generated indicates that *cqm1*_*REC-2*_ can be selected in field populations, although its low frequency and distribution in Recife suggest that *cqm1*_*REC-2*_ presents a lower risk of selection compared to *cqm1*_*REC*_.

**Electronic supplementary material:**

The online version of this article (doi:10.1186/s13071-016-1347-2) contains supplementary material, which is available to authorized users.

## Background

*Lysinibacillus sphaericus* based biolarvicides have been used worldwide for mosquito control and in particular against larvae from the *Culex pipiens* complex and *Anopheles* spp, owing to the high susceptibility of these species to the bacterium and the prolonged persistence of *L. sphaericus* in mosquito breeding sites in urban environments [1–3]. Endemic lymphatic filariasis is still found within the Recife Metropolitan Area (RMA) and its causative agent, *Wuchereria bancrofti*, is transmitted by *Culex quinquefasciatus* in some districts [4, 5]. Successful trials for controlling this species have been carried out in the RMA using *L. sphaericus* applications in combination with other environmental management actions [6–8]. These biolarvicides have been shown to perform very well in controlling *C. quinquefasciatus* but their use should be monitored because of the risk of resistance selection in treated populations. The major insecticidal factor produced by *L. sphaericus* is the Binary (Bin) protoxin, synthesized inside crystalline inclusions within the bacterium [9]. *L. sphaericus* acts through the ingestion of crystals by the mosquito larvae and subsequent release of Bin protoxins that are processed within the insect’s midgut. The toxin’s active form binds to a *C. quinquefasciatus* receptor that has been identified as a 66-kDa α-glucosidase, named Cqm1. The receptor is attached to the midgut epithelium by a glycosylphosphatidylinositol (GPI) anchor and is essential for Bin toxin action [10, 11]. This mode of action, depending on the binding of one toxin to a single class of receptors, can be disrupted by alterations in the target site that results in high resistance levels [12].

Resistance to *L. sphaericus* has been recorded in laboratory-selected colonies [13, 14] and in exposed field populations [15–18] and the lack of the Cqm1 receptor on the midgut epithelium was the major cause for the failure of the Bin toxin to bind to the midgut [19–22]. Investigation of the molecular basis of resistance has led to the identification of eight resistance alleles for the *cqm1* gene, found in colonies or populations from different geographical origins in the USA, Brazil, France, and China [19, 23–27]. Seven of these have deletions or nonsense mutations that create premature stop codons, resulting in transcripts coding for truncated proteins without a GPI anchor. Four of these alleles have been described in *C. quinquefasciatus* from Recife and two in particular, named *cqm1*_*REC*_ and *cqm1*_*REC-2*_, were co-selected in the R2362 laboratory colony [24, 27]. This colony was selected after continuous exposure to *L. sphaericus* and attained a high level of resistance, which was associated with the lack of midgut-bound Cqm1 receptors [13, 22]. A 19-nucleotide deletion (nt1257 to 1275) is responsible for the *cqm1*_*REC*_ resistance phenotype [27], while a single nucleotide transition (G1324A) was found to characterize the *cqm1*_*REC-2*_ allele [24]. Both events create premature stop codons that prevent the expression of the midgut bound Cqm1 receptors. These alleles are recessively inherited and homozygous larvae for them are highly resistant to *L. sphaericus*, owing to the failure of Bin toxin to bind to the midgut epithelium [24].

The R2362 colony has been kept under laboratory conditions for more than 200 generations and *cqm1*_*REC*_ was originally described as the single allele associated with resistance in this colony. Subsequently, *cqm1*_*REC-2*_ was identified as a second resistance allele co-selected along with *cqm1*_*REC*_. Both alleles lead to a similar phenotype and they confer high resistance levels, although *cqm1*_*REC*_ was preferentially selected and displayed a significantly higher frequency in the long term (≈F_1_-F_160_). *cqm1*_*REC*_ was later replaced by *cqm1*_*REC-2*_, which became the most frequent allele, considering the genotypes for the *cqm1* locus found among larvae from this colony. The significant increase in *cqm1*_*REC-2*_ frequency in individuals from this colony seemed to be related to the amelioration of traits other than resistance, since they confer a similar phenotype, although this remains to be elucidated [24]. These alleles showed a singular evolution as this colony was maintained in a restricted environment without gene flow. The trajectory of the *cqm1*_*REC-2*_ allele within the R2362 colony raised questions concerning its presence in field populations and thus to its potential to be selected. After identification of *cqm1*_*REC*_ within the R2362 resistant colony, this allele was recorded in populations from the RMA [28]. The main aim of this study was to investigate, through DNA screening, the occurrence and frequency of *cqm1*_*REC-2*_ in *C. quinquefasciatus* in the RMA, in order to assess its importance for monitoring resistance to *L. sphaericus* in these areas. In addition, sequencing of *cqm1* genes was performed to identify polymorphisms within the field samples analyzed in this study which might shed light on the relation between the distinct resistance alleles.

## Methods

### Mosquito colonies

The *Culex quinquefasciatus* and *Aedes aegypti* colonies used in this study were maintained in the insectarium of the Aggeu Magalhães Research Center (CPqAM)-FIOCRUZ under controlled conditions (26 ± 1 °C, 70 % humidity, and 12:12 h L:D photoperiod). The larvae were reared in tap water and fed with cat food; adults were fed on a 10 % sucrose solution; females were also fed on chicken blood. The following *C. quinquefasciatus* colonies were used: 1) CqSF, here called S, a susceptible reference colony established from egg rafts collected in Recife, Brazil; 2) REC and 3) REC-2 colonies, composed of larvae homozygous for *cqm1*_*REC*_ and *cqm1*_*REC-2*_ alleles respectively that display high levels of resistance (≈100,000-fold) to *L. sphaericus* [24]; 4) *Ae. aegypti* RecLab reference colony, to obtain the genomic DNA used as a negative control for the PCR reactions.

### Mosquito populations

Five populations from the Recife Metropolitan Area (RMA) were investigated. Samples from one *L. sphaericus*-treated area (Água Fria-AFR) and four non-treated areas (Azeitona-AZE, Ipojuca-IPO, Jaboatão-JAB, Roda de Fogo-RFO) were screened. Eggs or larvae were collected as described by Chalegre et al. [23] and were maintained under insectarium conditions until the 4^th^ instar, when the larvae were harvested and stored at −70 °C until use.

### Multiplex PCR for *cqm1* allele detection

The DNA genomic samples used here included those available from a previous study using the same populations [23] in addition to DNA samples obtained from larvae of those populations kept at −70 °C that were subsequently extracted using DNAzol® (Invitrogen, Carlsbad, CA, USA). The present study used a multiplex PCR to amplify fragments and produce a profile of the possible genotypes for *cqm1*, *cqm1*_*REC*_ and *cqm1*_*REC−2*_ alleles [24]. The conditions of PCR reactions and primer sequences used are described in Chalegre et al. [24]. All samples that showed the amplification of fragments whose size was compatible with that of the diagnostic fragment (172 bp) from *cqm1*_*REC−2*_ allele were tested in a second PCR to confirm the amplification. These amplified fragments were then submitted to automatic sequencing in an ABI PRISM® 3100 Genetic Analyzer (Applied Biosystems) to confirm their identity.

### Cloning and *cqm1* sequencing

To identify the polymorphisms within the *cqm1* sequence, genomic DNA was extracted and PCR reactions were carried out with primers flanking the *cqm1* full length coding sequence using Platinum® *Taq*DNA Polymerase High Fidelity® (Invitrogen), as described [28]. The PCR fragments were cloned in pGEM®-T Easy (Promega) and submitted to automatic sequencing as described above. The alignment and assembly of the resulting nucleotide and amino acid sequences was achieved using the DNASTAR software package and manual refinement was carried out when necessary. The sequences obtained were compared to the *cqm1* reference sequence available in GenBank (DQ333335) and to other sequences described in the Results section.

## Results

**Fig. 1:**
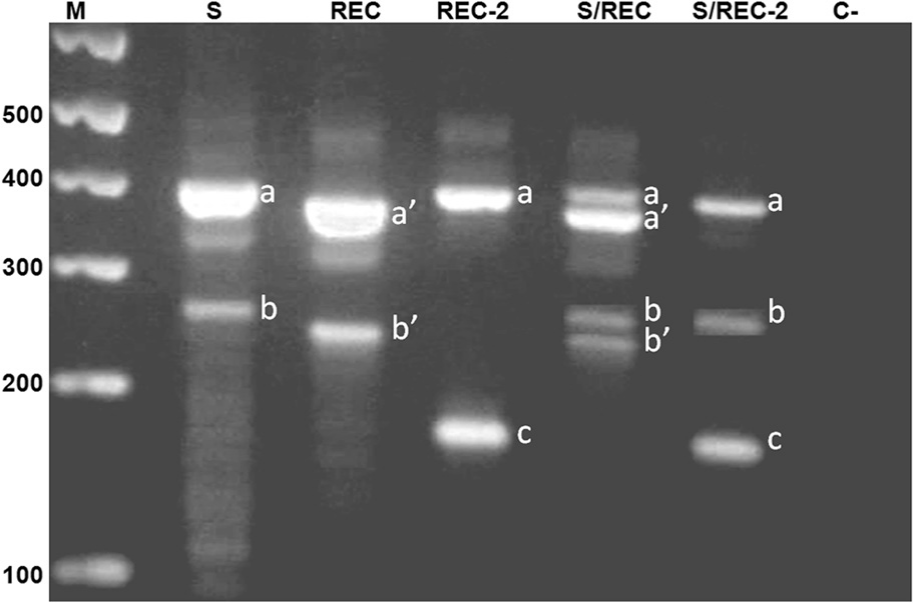
Fragments of *Culex quinquefasciatus cqm1* alleles amplified from larvae of susceptible (S) and *Lysinibacillus sphaericus*-resistant colonies (REC, REC-2). Profile of fragments in base pairs (bp): S (ab, 376-257 bp), REC (a’b’, 357-238 bp) and REC*-2* homozygous (ac, 376-172 bp); S/REC (aa’bb’) and S/REC-2 (abc) heterozygous; sample without DNA (C-). Molecular markers (M) in base pairs

A previous study assessing the frequency of *cqm1*_*REC*_ in field populations from the Recife Metropolitan Area (RMA) found this allele in all populations investigated [23, 28], confirming that it was already present in larvae samples used to establish the R2362 laboratory colony. This implies that the establishment of resistance did not require the selection of a novel spontaneous mutation arising within the colony. However, the lower frequency of *cqm1*_*REC-2*_ during the earlier phases of the selection process may indicate a novel resistance event, which appeared only after the colony was established. To evaluate this possibility, the presence of this allele was investigated in *C. quinquefasciatus* populations from the RMA that had previously tested positive for the occurrence of *cqm1*_*REC*_. The screening of *cqm1*_*REC-2*_ was performed using a multiplex PCR [24] whose association of primers can amplify fragments and define the various genotypes identified so far, as shown (Fig. 1), that are homozygous for the *cqm1*, *cqm1*_*REC*_ and *cqm1*_*REC-2*_ alleles (lanes 2, 3 and 4, respectively), and heterozygous for *cqm1/cqm1*_*REC *_and *cqm1*/*cqm1*_*REC-2*_ (lanes 5 and 6, respectively). In this assay, a control fragment (376–357 bp) can be amplified from the *cqm1* gene of all larvae, a diagnostic fragment (257–238 bp) is derived from the *cqm1* and *cqm1*_*REC*_ alleles alone and a third fragment (172 bp) is exclusively amplified in the presence of *cqm1*_*REC-2*_, whose forward primer was designed to match the nonsense mutation G1324A. Fragments amplified from *cqm1* and *cqm1*_*REC*_ can be distinguished because the *cqm1*_*REC*_ 19-nt deletion lies within this region and their corresponding products are slightly smaller. Fragments whose sizes were not compatible with those amplified from the *cqm1*_*REC*_ and *cqm1*_*REC-2*_ alleles were considered derived from the wild type *cqm1* gene*.*

**Fig. 2:**
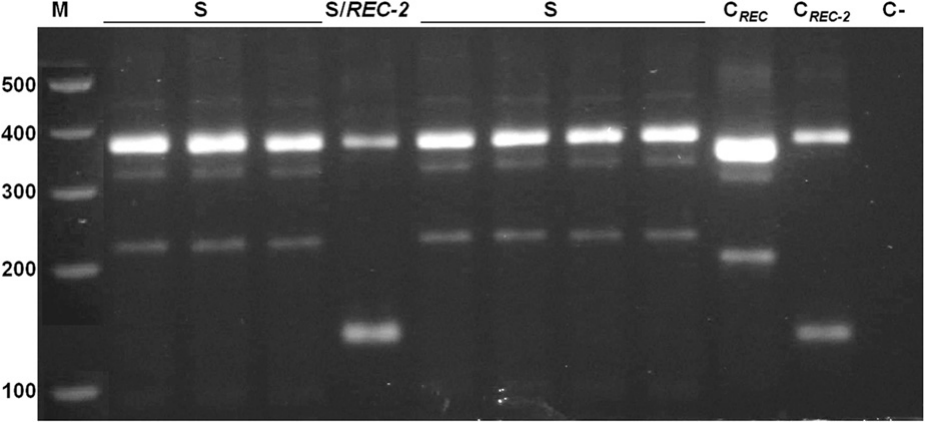
Fragments of *Culex quinquefasciatus cqm1* alleles amplified from Jaboatão field samples. *cqm1* homozygotes (S), *cqm1/cqm1*_*REC-2*_ heterozygote (S/REC-2), *cqm1*_*REC*_ (C_*REC*_) and *cqm1*_*REC-2*_ (C_*REC-2*_) internal positive controls for the respective alleles, samples without DNA (C-). Molecular markers (M) in base pairs

DNA samples of individual larvae from four non-treated and one *L. sphaericus* treated field populations were analyzed to investigate the presence of the *cqm1*_*REC-2*_ allele. Between 223 and 600 larvae per population were analyzed, comprising a total of 2,049 individuals. According to the diagnostic profile of the multiplex PCR, two heterozygous individuals carrying the *cqm1*_*REC-2*_ allele were found in the non-treated area of Jaboatão (Fig. 2). The identity of the corresponding diagnostic PCR fragments was assessed through sequencing and the G1324A transition was found in both samples. This allele was not detected in any other sample evaluated in this study (Table 1). The frequency of *cqm1*_*REC-2*_ in Jaboatão was 2 x 10^−3^ while this falls to 4.9 x 10^−4^ if all populations are considered, since it was found in samples from one among five populations that were analyzed in this study (Table 1). This new screening also detected the presence of the *cqm1*_*REC*_ allele, as expected, given the results of the previous screening carried out in these areas. In contrast to *cqm1*_*REC-2*_, *cqm1*_*REC*_ was detected in all populations sampled as heterozygous larvae and its frequencies ranged from 2 to 6 x 10^−3^ (Table 1).

**Table 1:** Genotypes for *cqm1*, *cqm1*_*REC*_ and *cqm1*_*REC-2*_ alleles detected in *Culex quinquefasciatus*

		Genotypes^b^			Frequency	
Sample^a^	No.	*cqm1/cqm1*	*cqm1/cqm1*_*REC*_	*cqm1/cqm1*_*REC-2*_	*cqm1*_*REC*_	*cqm1*_*REC-2*_
AZE	223	221	2	0	0.004	0
IPO	500	498	2	0	0.002	0
JAB	500	495	3	2	0.003	0.002
RFO	226	224	2	0	0.004	0
AFR	600	593	7	0	0.006	0
Total	2049	2031	16	2	3.9 × 10^−3^	4.9 × 10^−4^

^a^Larvae population samples from districts of Recife city (Brazil)

^b^Determined by multiplex PCR for the alleles analyzed

**Fig. 3:**
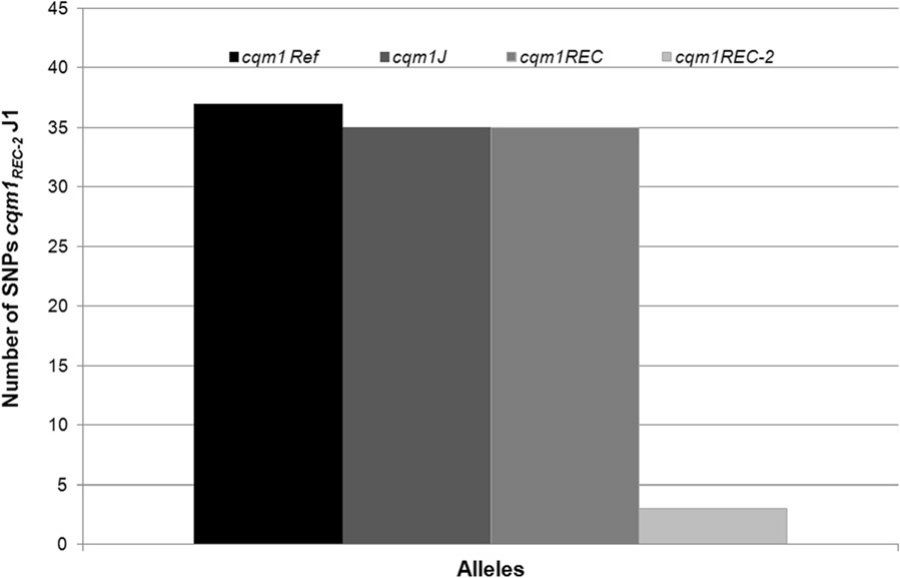
Number of single nucleotide polymorphisms (SNPs) between Jaboatão *cqm1*_*REC-2*_ sequence (*cqm1*_*REC-2*_ J1) versus other *cqm1* sequences. *cqm1* reference (Ref) (GeneBank DQ333335), *cqm1* from Jaboatão (J), *cqm1*_*REC*_ and *cqm1*_*REC-2*_, from the respective REC and REC-2 laboratory resistant colonies

To establish the relation of *cqm1*_*REC-2*_ alleles found in Jaboatão with other *cqm1* alleles investigated in this study, wild type or mutants, full-length sequences were obtained and, after amplification and sequencing, their polymorphisms were compared (Additional file 1: Table S1). The two sequences from the *cqm1*_*REC-2*_ alleles found in Jaboatão were identical except for a single nucleotide polymorphism (SNP) found at position 39. These sequences were then compared to the *cqm1*_*REC-2*_ allele cloned from larvae of the REC-2 resistant colony that are homozygous for *cqm1*_*REC-2*_ and only three additional SNPs distinguish the two Jaboatão alleles from the one found in the laboratory resistant colony (Additional file 1: Table S1). This identity level indicates that the *cqm1*_*REC-2*_ allele selected in the laboratory colony derived from field samples used to establish that colony. By contrast, a comparison between the Jaboatão (J1) *cqm1*_*REC-2*_ sequence and the *cqm1* reference sequence (Gene Bank DQ 333335) identified 37 SNPs between the two sequences and nine of those led to changes in amino acids, showing significant differences between these alleles, which seem to be unrelated to the resistance-causing mutation. When the Jaboatão (J1) *cqm1*_*REC-2*_ was compared with other sequences analyzed here, including *cqm1*_*REC*_, a similar number of 35 to 37 SNPs between these sequences were observed (Fig. 3). To understand these sequence differences and evaluate the range of polymorphisms seen within the coding sequence for this protein, an independent survey was conducted of the full-length *cqm1* gene sequences from another sample of fifteen *C. quinquefasciatus* larvae from Água Fria. The various *cqm1* alleles sequenced can be broadly assigned to two categories, defined as “variants” 1 and 2, based on nucleotide changes which lead to substitutions in a group of amino acid residues (Table 2). Sequences of these representative “variants” were then compared with those included in the present study and the data shown highlight the fact that Jaboatão (field) and laboratory *cqm1*_*REC-2*_ copies show the same polymorphisms and that these are distinct from those seen in *cqm1*_*REC*_. When compared with the “variant” *cqm1* alleles, this data indicates that *cqm1*_*REC*_ is derived from an allele linked to “variant 1” of *cqm1* alleles, while *cqm1*_*REC-2*_ is associated with “variant 2” (Table 2).

**Table 2:** Polymorphisms found in protein sequences coded by *cqm1* alleles

	Amino acid position																	
Alelle^a^	3	8	15	26	27	141	178	246	335	336	430	439	450	466	529	530	541	556
Ref	Pro	Ser	Met	Asp	Ser	Ala	Thr	Ser	Ser	Ser	Thr	Ala	Pro	His	Asn	Ser	Gly	Thr
AFR1	Pro	Gly	Met	^b^	^b^	Ala	Thr	Ser	^b^	^b^	Thr	Thr	Pro	His	Lys	^b^	Gly	Met
AFR2	Thr	Ser	Thr	^b^	^b^	Val	Gln	Pro	^b^	^b^	Ser	Ala	Ser	Tyr	Asn	^b^	Ser	Thr
JAB	Pro	Ser	Thr	Val	Ser	Val	Gln	Pro	Asn	Ser	Thr	Ala	Pro	His	Asn	Ser	Gly	Thr
REC	Pro	Ser	Met	Asp	Ser	Ala	Thr	Ser	Ser	Ser	Thr	Ala	Pro	His	Asn	Ser	Gly	Thr
REC2	Thr	Ser	Thr	Asp	Leu	Val	Gln	Pro	Ser	Pro	Ser	Ala	Ser	Tyr	Asn	Pro	Ser	Thr
REC2 J1	Thr	Ser	Thr	Asp	Ser	Val	Gln	Pro	Ser	Ser	Ser	Ala	Ser	Tyr	Asn	Ser	Ser	Thr
REC2 J2	Pro	Ser	Thr	Asp	Ser	Val	Gln	Pro	Ser	Ser	Ser	Ala	Ser	Tyr	Asn	Ser	Ser	Thr

^a^ Ref: *cqm1* sequence (GeneBank DQ333335); AFR1/AFR2: *cqm1* types 1 and 2 from Agua Fria; JAB: *cqm1* from Jaboatão; REC and REC-2: *cqm1*_*REC*_ and *cqm1*_*REC-2*_ from the respective laboratory resistant colonies; REC2 J1/REC2 J2: *cqm1*_*REC-2*_ from two Jaboatão individuals

^b^ Not determined

## Discussion

The *cqm1*_*REC-2*_ allele that confers high resistance to *L. sphaericus*, originally identified in the *C. quinquefasciatus* R2362 laboratory selected colony, was found also to occur in the field populations of Recife. *cqm1*_*REC-2*_ was co-selected with the *cqm1*_*REC*_ allele in a colony that has been maintained under laboratory conditions for a period corresponding to more than 200 generations [24]. The earliest detection of *cqm1*_*REC-2*_ in R2362 larvae was in F_35_, when it was found with a frequency of 0.21, contrasting with the frequency of 0.79 for *cqm1*_*REC*_. From F_182_ onwards, however, *cqm1*_*REC-2*_ replaced *cqm1*_*REC*_ as the most frequent allele conferring resistance in the colony, with an average frequency of 0.69. The detection of *cqm1*_*REC-2*_ in a population suggests that it was present in field larvae samples used to establish the R2362 laboratory colony, rather than being derived from a mutation that occurred during the selection process [13]. Its detection in the field also demonstrates that its frequency can be tracked in populations exposed to treatments, unlike some resistance alleles that were identified in colonies but were not detected in field populations and whose risk of selection could not be directly assessed [29]. Its frequency therefore can and should be monitored in areas exposed to *L. sphaericus* treatment.

*cqm1*_*REC-2*_ is the fourth *cqm1* allele associated with resistance that was found to occur in *C. quinquefasciatus* populations from the RMA, along with *cqm1*_*REC,*_*cqm1*_*REC-16*_ and *cqm1*_*REC-25*_ [23, 28]. *cqm1*_*REC-2*_ showed a limited distribution, being detected in one out of the five populations surveyed, in contrast to *cqm1*_*REC*,_ which was found in all eight populations investigated to date [23, 28]. Likewise, the *cqm1*_*REC-16*_ and *cqm1*_*REC-25*_ alleles, identified in field populations only, also showed a limited distribution and frequency, when compared to *cqm1*_*REC*_ [23, 28]. Neither was *cqm1*_*REC-2*_ found in an *L. sphaericus*-treated population whose conditions could be considered propitious for the detection of resistance alleles. The data obtained from the screening of these resistance *cqm1* alleles concur with previous studies of resistance alleles to *Bacillus thuringiensis* (Bt) Cry toxins, which are present in populations that have not been previously exposed to control agents [30–32]. This scenario of pre-adaptation was also found for chemical insecticides and one illustrative case was the detection of esterase genes conferring organophosphate resistance found in *Lucilia cuprina* specimens collected before the introduction of synthetic insecticides on a global scale [33]. A recent study of *Helicoverpa* spp populations from Australia showed genes conferring resistance to the vegetative insecticidal toxins (Vips) also produced by Bt, at frequencies between 0.008 and 0.027. These mutation rates were higher than expected, given that the samples were analyzed before the deployment of Vip-expressing plants that were intended to manage the resistance to Cry toxins expressed in Bt-plants [34]. The presence of the resistance *cqm1* alleles in the non-treated populations also demonstrates their potential to respond to selection pressure. This set of studies has shown that a database of susceptibility to available insecticides and pre-existing mutations may be a valuable tool in guiding the choice of larvicides to be introduced in control programs and for monitoring the rise of resistance [35].

The sequence analysis of the *cqm1*_*REC-2*_ alleles found in Jaboatão provided evidence that can be used to track the possible origin of this allele. The results generated corroborate the hypothesis that the *cqm1*_*REC-2*_ selected in the R2362 colony derived from field population samples used to establish this colony. The analysis of single nucleotide polymorphisms (SNPs) showed that field and laboratory copies of *cqm1*_*REC-2*_ are almost identical, while these are clearly less related to *cqm1* and *cqm1*_*REC*_ sequences. All SNPs observed in the *cqm1*_*REC*_ sequence, for instance, were found in the *cqm1* reference sequence, which suggests a common origin, different from that observed for *cqm1*_*REC-2*_. Likewise, the polymorphism analysis at the protein sequence level revealed that the resistance alleles, *cqm1*_*REC*_ and *cqm1*_*REC-2*_, are derived from different *cqm1* variants found in field populations. During the evolution of the R2362 colony, the *cqm1*_*REC-2*_ allele showed the capacity to replace *cqm1*_*REC*_ after its initial co-selection and a lower frequency in this colony. This finding suggested that *cqm1*_*REC-2*_ may be associated with fitness advantages and could potentially be better established in field populations and more easily selected if these are subjected to selection pressure through *L. sphaericus* treatments. The data from the present study show that the *cqm1*_*REC-2*_ allele does occur in field populations, but its limited distribution and lower frequency, compared to *cqm1*_*REC*_ in the RMA, make its selection less likely.

## Conclusions

The results of the present study therefore show the origin and occurrence of both resistance alleles in field populations of the RMA with a limited representation for *cqm1*_*REC-2*_ in terms of frequency and distribution, compared to *cqm1*_*REC*_. This scenario suggests selection to be less likely when resistance management strategies deployed in exposed mosquito populations are considered. These resistant alleles were found to be associated with two distinct wild-type *cqm1* variants found in field populations, suggesting distinct origins. Multiple resistance *cqm1* alleles were found to occur in the RMA and these may play distinct roles for selection. These factors should be taken into account when designing resistance management strategies.

## Additional file

Additional file 1: Table S1. Polymorphisms found on the full-length nucleotide sequence of *Culex quinquefasciatus cqm1* alleles*.cqm1*_*REC-2*_ from two Jaboatão larvae (J1, J2), *cqm1*_*REC-2*_ from a reference resistant colony (REC-2), *cqm1* from a reference (Ref) sequence (GenBank DQ333335), *cqm1* from Jaboatão larvae (J), *cqm1*_*REC*_ a from a reference resistant colony (REC). * Position corresponding to a 19-nt deletion, **non sense mutation, *** not determined. (PDF 54 kb)
