# Supplementary material for: A new allele conferring resistance to Lysinibacillus sphaericus is detected in low frequency in Culex quinquefasciatus field populations
Source: Parasit Vectors. 2016 Feb 4;9:70. doi: 10.1186/s13071-016-1347-2 (PMC4743420; doi:10.1186/s13071-016-1347-2)
Supplement: Additional file 1: Table S1. — Polymorphisms found on the full-length nucleotide sequence of Culex quinquefasciatus cqm1 alleles.cqm1 REC-2 from two Jaboatão larvae (J1, J2), cqm1 REC-2 from a reference resistant colony (REC-2), cqm1 from a reference (Ref) sequence (GenBank DQ333335), cqm1 from Jaboatão larvae (J), cqm1 REC a from a reference resistant colony (REC). * Position corresponding to a 19-nt deletion, **non sense mutation, *** not determined. (PDF 54 kb) [file 13071_2016_1347_MOESM1_ESM.pdf]

**Table S1.** Polymorphisms found on the full-length nucleotide sequence of *Culex quinquefasciatus cqm1* alleles. $cqm1_{REC\text{-}2}$ from two Jaboatão larvae (J1, J2), $cqm1_{REC\text{-}2}$ from a reference resistant colony (REC-2), *cqm1* from a reference (Ref) sequence (GenBank DQ333335), *cqm1* from Jaboatão larvae (J), $cqm1_{REC}$ a from a reference resistant colony (REC). * Position corresponding to a 19-nt deletion, **non sense mutation, *** not determined.

| | $cqm1_{REC\text{-}2}$ | | | *cqm1* | | $cqm1_{REC}$ | | $cqm1_{REC\text{-}2}$ | | | *cqm1* | | $cqm1_{REC}$ |
|---|---|---|---|---|---|---|---|---|---|---|---|---|---|
| Position | J1 | J2 | REC-2 | Ref | J | REC | Position | J1 | Jab2 | REC-2 | Ref | Jab | REC |
| 39 | A | C | A | C | C | C | 1172 | T | T | T | C | C | C |
| 76 | C | C | C | T | C | T | 1226 | C | C | C | C | T | C |
| 109 | A | A | A | A | T | A | 1229 | A | A | A | A | G | A |
| 112 | C | C | T | C | C | C | 1232 | G | G | G | G | A | G |
| 152 | C | C | C | T | C | T | 1244 | C | C | C | C | T | C |
| 188 | A | A | A | T | A | T | 1253 | A | A | A | A | G | A |
| 224 | T | T | T | C | C | C | 1265 | G | G | G | G | A | * |
| 225 | C | C | C | T | T | T | 1271 | G | G | G | G | T | * |
| 278 | T | T | T | G | T | G | 1320 | T | T | T | A | A | A |

| | | | | | | | | | | | | | |
|---|---|---|---|---|---|---|---|---|---|---|---|---|---|
| 284 | G | G | G | A | A | A | 1324** | A | A | A | G | G | G |
| 287 | T | T | T | C | C | C | 1343 | C | C | C | C | T | C |
| 296 | C | C | C | T | T | T | 1346 | T | T | T | C | C | C |
| 383 | C | C | C | G | G | G | 1361 | A | A | A | A | G | A |
| 401 | C | C | C | T | C | T | 1373 | A | A | A | G | A | G |
| 425 | T | T | T | C | T | C | 1380 | T | T | T | C | C | C |
| 454 | T | T | T | C | T | C | 1421 | G | G | G | T | G | T |
| 470 | T | T | T | C | T | C | 1428 | T | T | T | C | C | C |
| 494 | T | T | T | C | T | C | 1487 | T | T | T | A | T | A |
| 564 | C | C | C | A | C | A | 1488 | C | C | C | T | T | T |
| 565 | A | A | A | C | A | C | 1541 | C | C | C | C | T | C |
| 641 | T | T | T | G | G | G | 1568 | T | T | T | A | A | A |
| 768 | C | C | C | T | C | T | 1601 | T | T | T | C | T | C |
| 875 | G | G | G | G | A | G | 1620 | T | T | C | T | T | T |
| 1036 | G | G | G | G | A | G | 1631 | A | A | A | G | G | G |

| | | | | | | | | | | | | | |
|---|---|---|---|---|---|---|---|---|---|---|---|---|---|
| 1038 | T | T | C | T | T | T | 1634 | T | T | T | G | G | G |
| 1103 | T | T | T | T | C | T | 1637 | A | A | A | C | C | C |
| 1109 | C | C | C | C | T | C | 1653 | A | A | A | G | G | G |
| 1112 | G | G | G | G | A | G | 1766 | *** | *** | C | T | *** | T |
